# Prevalence, density and predictors of malaria parasitaemia among ill young Nigerian infants

**DOI:** 10.11604/pamj.2021.40.25.30172

**Published:** 2021-09-09

**Authors:** Oluwatobi Faith Folarin, Bankole Peter Kuti, Akibu Oyeku Oyelami

**Affiliations:** 1 Department of Paediatrics, Wesley Guild Hospital, Ilesa, Nigeria,; 2 Department of Paediatrics and Child Health, Obafemi Awolowo University, Ile-Ife, Nigeria

**Keywords:** Low birth weight, malaria, parasitaemia, young infants

## Abstract

**Introduction:**

infants in the first six months of life are relatively protected from malaria. Emerging reports from endemic regions however are showing increasing malaria susceptibility in this age group. This study set out to determine the prevalence, parasite density and predictive factors for malaria parasitaemia in ill young infants at the Wesley Guild Hospital (WGH), Ilesa, Nigeria.

**Methods:**

ill infants aged one to six months were consecutively recruited over an 11-month period in a hospital based cross-sectional study. History of illness, sociodemographic and perinatal history were obtained; clinical examination and results of venous blood for thick and thin film malaria parasite examinations were recorded and analyzed.

**Results:**

the mean (SD) age of the 350 infants was 3.4 (1.6) months with male: female (M: F) of 1.2: 1. The prevalence of malaria parasitaemia (all plasmodium falciparum) was 19.1% while parasite density ranged from 24.0 to 400,000 parasites/µl, median (IQR) 900 (250-4,588)/µl. Sixteen (4.6%) had heavy malaria parasitaemia (>5000/µl). Low social class (OR=2.457; 95%CI 1.404-4.300; p=0.002), suboptimal antenatal care (OR=2.226; 95%CI 1.096-4.522; p=0.027), low birth weight infants (OR=4.818; 95%CI 2.317-10.018; p=<0.001) and injudicious use of haematinics (OR=3.192; 95%CI1.731-5.886; p=<0.001) were predictors of malaria parasitaemia among the infants.

**Conclusion:**

one-in-five ill young infants had malaria parasitaemia with heavy parasitaemia in 23.8% of infected infants. Malaria parasitaemia was associated with modifiable factors, high index of suspicion in endemic region and optimal maternal and child care services may assist to reduce the burden of malaria in this age group.

## Introduction

Malaria is an acute febrile illness in humans which can sometimes be fatal especially in young children [1]. It is an infectious disease caused by the presence and activities of plasmodium protozoa in human blood [1]. These Plasmodium species are transmitted from one infected human to another by the vector, female anoepheles mosquito [1]. Malaria is a major cause of morbidity and mortality especially in sub-Saharan Africa [2]. Globally, over 90 percent of deaths due to malaria infection occur in the African sub-region [2]. Nigeria alone accounts for about one-fifth of these global deaths [2]. Children under five years of age in Africa are most affected by malaria-related morbidity and mortality [2]. However, young infants (aged one to six months) have over the years been perceived to be relatively protected from malaria and its complications [2, 3].

Young infants have remarkably high level of foetal haemoglobin which makes their red blood cells to be relatively resistant to being parasitized by the plasmodium parasite [1]. Also, the reduced amount of para-amino-benzoic acid (PABA) in the red blood cells of young infants is also protective from malaria as the parasite requires PABA as substrate for rapid proliferation [1, 4]. Additionally, antibodies and lactoferrin from breastmilk [5], as well as maternal-derived malarial antibodies transferred to the baby via the placenta help to protect these infants from malaria infection [6]. Furthermore, the usual practice of wrapping babies with clothes and keeping them indoors also protects them from mosquito bites [7]. These factors have been recognised as being protective against malaria in young infants particularly in endemic regions like Nigeria [6, 7].

There appears to be a gradual change in the current epidemiology of malaria infection to include young infants who were previously believed to be relatively protected [8]. Different studies in Africa, have reported varying but significant prevalence of malaria parasitaemia among young infants. The reported prevalence ranged from 3.7% from The Gambia [9], 10.2% from Benin republic [9], 21.7% from Guinea [9] and 27.1% from Lagos, Nigeria [8]. Most of these studies reported predominantly light parasite densities among the infected young infants [8, 9]. For instance, Afolabi *et al*. [8] in Lagos, Nigeria reported that 80% of the young infants with malaria parasitaemia had parasite densities between 1-500 parasites per microliter with the mean parasite density being 202.5 parasites per microliter.

The observed changing trends in the occurrence of malaria infection among young infants who are hitherto believed to be protected from the infections may be due to a number of factors. These include the increasing virulence of the plasmodium parasites viz-a-viz emerging resistance of the parasite to commonly used antimalarials such as sulphadoxine-pyrimethamine used for intermittent preventive therapy (IPT) in pregnancy [10]. Also, emerging resistance of some species of the female anopheles mosquitoes to insecticides has been reported [11]. Additionally, some studies have linked low rate of exclusive breastfeeding [12], low compliance with the recommended three doses of IPT in pregnancy [12], poor environmental sanitation [8] and overcrowding especially in urban slums [8] to increased malaria parasitaemia in young infants.

The sociodemographic, perinatal and environmental factors associated with malaria parasitaemia in young infants may vary from one region to another. Recognizing these factors can facilitate appropriate intervention to reduce the burden of malaria in this age group. Also, prompt diagnosis and treatment of malaria in this age group is very important to reduce malaria-related childhood morbidity and mortality particularly in endemic regions. This study therefore set out to determine the prevalence of malaria parasitaemia in young infants presenting with ill-health at the children welfare clinic (CWC) and children emergency ward (CEW) of the Wesley Guild Hospital (WGH), Ilesa, south-west Nigeria; to characterise the parasite density and identify the predictive factors associated with malaria parasitaemia among these infants.

## Methods

**Study design and setting:** this was a hospital-based cross-sectional study carried out over an 11-month period (September 2018 to July 2019). The study was carried out at the CWC and CEW of the WGH, Ilesa, south-west Nigeria. The WGH is a tertiary arm of the Obafemi Awolowo University Teaching Hospitals Complex (OAUTHC), Ile-Ife, Nigeria. The CWC attends to children with different complaints and ailments while children in need of emergency interventions are admitted to the CEW. Ilesa (long. 7037´N, lat. 4043´E) is a semi-urban city, located in the tropical rain forest belt of Nigeria. It is located about 377 meters above the sea level and about 250 km north-east of Lagos the commercial capital of Nigeria [13]. The city experiences dry and rainy (wet) season which lasts between the months of March and November. Annual rainfall is heavy with mean rainfall varying between 1140 mm to 1320 mm [14]. Hence study location is holo-endemic for malaria which is reported all year round in the city.

**Study population:** all ill young infants (aged one to six months) who presented at the WGH, Ilesa (CWC and CEW) and whose mothers/caregivers gave consent were consecutively recruited until the minimum sample size of 350 was attained. Ill health in this study included complaints like fever, hypothermia, refusal to feed, cough, catarrh, difficulty in breathing, vomiting, convulsion and pallor.

**Exclusion criteria:** prior use of antimalaria or any drug combination containing antimalarial, young infants diagnosed with surgical conditions.

**Study size:** the minimum sample size of 350 ill young infants was determined using the Leslie Fisher´s formula [15]. Assumptions made included an estimated prevalence of ill young infants with malaria parasitaemia from a previous study as 27.1% [8], alpha value of 5% at 95% confidence interval (CI) and p-value of 0.05.

**Data collection:** caregivers/parents who consented were verbally interviewed and their responses recorded in prepared questionnaires. Information obtained included age (in months), sex, birth weight and feeding history (including the use of pre-lacteal feeds or exclusive breastfeeding). History of routine use of iron/folic acid supplements (haematinics) in the infants was also obtained. Maternal data included maternal age, place of antenatal care and delivery (orthodox health facilities include hospitals and maternity centres while unorthodox places were mission and traditional birth centres) and use of intermittent preventive therapy for malaria during pregnancy. Also, maternal parity which included primiparous (one childbirth), multiparous (2-4 childbirths) and grand-multiparous (≥five childbirths) was also obtained. Parental educational status, occupation, income, presence of window nets (torn or intact), use of insecticidal treated bed nets, indoor residual spraying of insecticide and presence of cymbopogon citrates (lemon grass) with mosquito repellent properties [16] around the house were also obtained. Socioeconomic status of the parents was assessed using the Ogunlesi *et al*. [17] classification based on the highest educational qualification, occupation and income of both parents. The upper class refers to socioeconomic classes I & II, middle class is socioeconomic class III and socioeconomic classes IV and V belong to the lower class. The study participants were examined and clinical findings documented. Laboratory investigation was done by collecting two millilitres of peripheral venous blood using aseptic techniques from each ill young infant and this was used for preparing thick and thin blood films and stained with 10% Giemsa stain for malaria parasite density and specie recognition respectively using standard methods [18]. The parasite density was determined microscopically by counting the number of parasites per 200 white blood cells visualized per high power field and this was extended to 500 white blood cells before a slide was declared negative. The parasite density per microliter was derived from the number of parasites per 200 white blood cells multiplied by 8,000. This is based on the assumed count of 8,000 white blood cells per microliter [18]. Parasite density (parasite/microliter) = (number of parasites counted x 8000) / 200.

The malaria parasite density was classified into the two different parasite classes according to the standard recommendation of Spencer [19] as heavy parasitaemia (>5000parasites/µl) and light parasitaemia (≤5000parasites/µl). Two experienced microscopists were involved in the study and they read the slides independent of each other. The final parasite density of the positive slides was an average of the values obtained by both scientists. The conflicting microscopy results were crosschecked by a third microscopist (WHO certified) and his results were taken as the final result for such slides.

**Statistical analysis:** this was done using the Statistical Package for Social Sciences (SPSS) version 20.0 (IBM, USA). The prevalence of malaria parasitaemia among the infants was determined by finding the proportion of the infants with malaria parasitaemia over the total number of study participants. Categorical variables like sex, age groups and social class were summarised using percentages, while continuous variables like age and parasite count was summarised using the mean (standard deviation) or median (interquartile range) as appropriate. Categorical variables were analysed using Pearson´s Chi-square test and likelihood ratio as appropriate. Multivariate logistic regression analysis was used to determine the independent risk factors for the dichotomized outcome (presence vs absence of malaria parasitaemia). This was done by identifying risk factors that were statistically significant using the Pearson´s Chi-square test (bivariate analysis) and these identified factors were loaded step-wisely into the multivariate logistic regression model. Results were interpreted as odd ratio and 95% confidence interval given. Statistical significance was recorded at p-value <0.05. The inter-observer concordance of the microscopists who read the malaria parasite slides was determined using the inter-observer percentage agreement.

**Ethical consideration:** this study was approved by the Ethics and Research Committee of the OAUTHC Ile-Ife with protocol number ERC/2018/04/01. Written informed consent was also obtained from parents and/or accompanying care givers of the infants.

## Results

**Background characteristics:** three hundred and fifty young infants were recruited for the study over an eleven-month period. Two hundred and eighty-nine (82.6%) infants were recruited via the CWC and managed on out-patient basis while 61 (17.4%) were managed as in-patient at the CEW. There was no missing data in this study. All study participants had their parasite density results documented and analyzed.

Sociodemographic characteristics of the study participants: these are highlighted in Table 1. The mean (SD) age of the infants was 3.4 (1.6) months, with 54.6% in the age range one to three months. One hundred and ninety-three (55.1%) were males giving a male: female ratio of 1.2: 1. The study was enriched with infants whose parents reside in Ilesa (84.6%), from low socioeconomic class (47.4%) and of Yoruba ethnicity (89.4%). Housing factors: the majority (89.7%) of the study participants claimed to have window nets, but only 170 (48.6%) sleep under bed net, also use of insecticide spray (40.3%), net at the main door (29.1%) and insect repellent plants like lemon grass were uncommon (10.6%) (Table 1).

**Table 1:** association between socio-demographic and housing characteristics and malaria parasitaemia among the study participants

Socio-demographic factors	Malaria parasitaemia n=67 (%)	No Malaria parasitaemia n=283 (%)	Total n=350	x^2^	p-value
**Gender**					
Male	36 (53.7)	157 (55.5)	193	0.067	0.796
Female	31 (46.3)	126 (44.5)	157		
**Age group (months)**					
1-<4	38 (56.7)	153 (54.1)	191	0.154	0.695
4-6	29 (43.3)	130 (45.9)	159		
**Socioeconomic classification**					
Low	44 (65.7)	122 (43.1)	166	11.473	0.003
Middle	17 (25.3)	130 (45.9)	147		
Upper	6 (9.0)	31 (11.0)	37		
**Ethnicity**					
Yoruba	55 (82.1)	258 (91.2)	313	5.023	0.170*
Hausa	5 (7.5)	8 (2.8)	13		
Ibo	3 (4.5)	10 (3.5)	13		
Others	4 (5.9)	7 (2.5)	11		
**Place of residence**					
Within Ilesa	51 (76.1)	245 (86.6)	296	4.537	0.033
Outside Ilesa	16 (23.9)	38 (13.4)	54		
**Window nets**					
Yes	52 (77.6)	262 (92.6)	314	13.152	<0.001
No	15 (22.4)	21 (7.4)	36		
Condition of window nets*					
Torn window nets	8 (11.9)	37 (13.1)	45	0.042	0.837
Window nets intact	44 (65.7)	225 (79.5)	269		
No window nets	15 (22.4)	21 (7.4)	36		
**Use of bed-net**					
Yes	27 (40.3)	143 (50.5)	170	2.270	0.132
No	40 (59.7)	140 (49.5)	180		
**Use of insecticide spray**					
Yes	29 (43.3)	112 (39.6)	141	0.310	0.578
No	38 (56.7)	171 (60.4)	209		
**Net on main door**					
Yes	13 (19.4)	89 (31.4)	102	4.737	0.940
No	54 (80.6)	194 (68.6)	249		
**Use of haematinics**					
Yes	26 (38.8)	42 (14.8)	68	19.876	<0.001
No	41 (61.2)	241 (85.2)	282		
**Lemon grass around the house**					
Yes	9 (13.4)	28 (10.0)	37	0.718	0.397
No	58 (86.6)	255 (90.0)	313		


The figures in parentheses are percentages along each column * likelihood ratio applied *36 of the study participants did not have window nets

Maternal and neonatal history: the mean (SD) maternal age was 29.3 (5.8) years which ranged from 15.0 to 56.0 years. Teenagers (2.9%) and grand-multiparity (2.8%) were few among the participating mothers (Table 2). Most of the mothers (89.7%) had at least secondary education, had ANC (95.4%), use IPT in pregnancy (72.0%) and delivered their babies in orthodox places of delivery (86.0%). The majority (69.7%) of the infants were delivered per vaginam, and had normal birth weight (Table 2). Although, mothers alluded to breastfeeding their babies exclusively (60.3%), the use of prelacteal feeds (57.1%) was also common (Table 2). Clinical features at presentation: these are highlighted in Table 3. Fever was the most common clinical feature reported in 266 (76.0%) of the infants. Pallor (2.3%) and convulsions (1.4%) were rare.

**Table 2:** maternal and neonatal factors as related to malaria parasitaemia

Maternal factors	Malaria parasitaemia N=67(%)	No malaria parasitaemia N=283(%)	Total N=350	x^2^	p-value
**Mother's age (years)**					
<20	1 (1.5)	10 (3.5)	11	4.360*	0.113
20-35	61 (91.0)	227 (80.2)	288		
>35	5 (7.5)	46 (16.3)	51		
**Level of education**					
Postsecondary	25 (37.3)	164 (58.0)	189	11.170*	0.011
Secondary	30 (44.8)	95 (33.5)	125		
Primary	8 (11.9)	19 (6.7)	27		
No formal education	4 (6.0)	5 (1.8)	9		
**Parity**					
Primiparous	18 (26.8)	101 (35.7)	119	2.425*	0.297
Multiparous	46 (68.7)	174 (61.8)	221		
Grand multiparous	3 (4.5)	7 (2.5)	10		
**Antenatal care**					
Yes	60 (89.6)	274 (96.8)	334	6.559	0.010
No	7 (10.4)	9 (3.2)	16		
**IPT use in pregnancy**					
Yes	37 (55.2)	215 (76.0)	252	11.568	0.001
No	30 (44.8)	68 (24.0)	98		
**Place of antenatal care^#^**					
Orthodox facilities	53 (79.2)	258 (91.2)	311	2.607	0.106
Unorthodox places	7 (10.4)	16 (5.7)	23		
No antenatal care	7 (10.4)	9 (3.1)	16		
**Place of delivery**					
Orthodox facilities	50 (74.6)	251 (88.7)	301	8.902	0.003
Unorthodox places	17 (25.4)	32 (11.3)	49		
**Mode of delivery**					
Per vaginam	51 (76.1)	193 (68.2)	244	2.180	0.336
Caesarean section	16 (23.9)	90 (31.8)	106		
Birth weight categories**					
LBW	21 (31.3)	37 (17.1)	58	21.024*	<0.001
NBW	17 (25.4)	166 (76.9)	183		
Macrosomic	2 (3.0)	13 (6.0)	15		
No record	27	67	94		
Exclusive breastfeeding	28 (41.8)	183 (64.7)	211	11.838	0.001
No	39 (58.2)	100 (35.3)	139		
Pre-lacteal feeds	30 (44.8)	170 (60.1)	200	5.180	0.023
No	37 (55.2)	113 (39.9)	150		

* Likelihood ratio applied; the figures in parenthesis are percentages along the columns; ^#^16-mothers did not have receive antenatal care; **birthweight of 94 babies were not recorded; LBW-low birth weight; NBW-normal birth weigh

**Table 3:** presenting clinical factors and malaria parasitaemia

Clinical factors	Malaria parasitaemia N=67 (%)	No malaria parasitaemia N=283 (%)	Total N=350	x^2^	p-value
**Fever**					
Yes	55 (82.1)	211 (74.6)	266	1.685	0.194
No	12 (17.9)	72 (25.4)	84		
**Fast breathing**					
Yes	12 (17.9)	37 (13.1)	49	1.052	0.305
No	55 (82.1)	246 (86.9)	301		
**Vomiting**					
Yes	6 (9.0)	20 (7.1)	26	0.281	0.596
No	61 (91.0)	263 (92.9)	324		
**Poor feeding**					
Yes	4 (6.0)	11 (3.9)	15	0.178	0.673*
No	63 (94.0)	272 (96.1)	335		
**Diarrhoea**					
Yes	1 (1.5)	12 (4.2)	13	0.504	0.478*
No	66 (98.5)	271 (95.8)	337		
**Excessive crying**					
Yes	3 (4.5)	10 (3.5)	13	0.000	0.993*
No	64 (95.5)	273 (96.5)	337		
**Pallor**					
Yes	8 (11.9)	0 (0)	8	29.442	<0.001*
No	59 (88.1)	283 (100)	342		
**Convulsion**					
Yes	1 (1.5)	4 (1.4)	5	0.000	1.000*
No	66 (98.5)	279 (98.6)	345		

The figures in parenthesis are percentages across the columns; *Yates correction applied

**Prevalence and density of malaria parasitaemia in the infants:** the prevalence of malaria parasitaemia among the recruited infants was 19.1%. The median (IQR) for malaria parasite density was 900 (250 - 4,588)/µl which ranged from 24.0 to 400,000 parasites/µl. Sixteen (4.6%) infants had heavy parasitaemia, however, heavy parasitaemia was observed in 16 (23.8%) of 67 infants with parasitaemia. All the malaria parasites identified were of the plasmodium falciparum specie. The inter-observer percentage agreement between the first two microscopists that independently read the slides was 54.1%. Therefore, a third microscopist (WHO certified) separately examined the slides and resolved the disparity.

**Association between sociodemographic characteristics and malaria parasitaemia:** significantly, higher proportion of the infants whose parents reside outside Ilesa had malaria parasitaemia compared to those within Ilesa (23.9% vs 13.4%; x^2^=4.537, p=0.033). Likewise, parental low socioeconomic class predispose the infants to malaria parasitaemia (Table 1).

**Housing factors and malaria parasitaemia:** Table 1 shows the details of the relationship between housing factors and malaria parasite status of study participants. Absence of window nets was significantly associated with the detection of malaria parasitaemia in the infants.

**Use of haematinics (iron/folic acid supplements) and malaria parasitaemia:** the routine use of haematinics was significantly associated with the detection of malaria parasitaemia in the infants. Higher proportion of infants who had haematinics h ad malaria parasitaemia compared to those without malaria parasitaemia (38.8% vs 14.8%; x^2^=19.876, p=0.001).

**Maternal and perinatal history as related to malaria parasitaemia in the infants:** Table 2 highlights the association between the presence of malaria parasitaemia in the infants and maternal and perinatal history. Low maternal education (less than secondary education), lack of ANC, delivery in unorthodox places and failure to use IPT were significantly associated with malaria parasitaemia among the infants. Likewise, low birth weight, use of prelacteal feeds and lack of exclusive breastfeeding were associated with malaria parasitaemia.

**Presenting clinical features and malaria parasitaemia:** details about the presenting clinical features of the study participants as related to the presence or absence of malaria parasitaemia are shown in Table 3. All the babies with pallor had associated malaria parasitaemia. No other presenting feature was significantly associated with the detection of malaria parasitaemia in the infants.

**Predictors of malaria parasitaemia among the infants using multivariate logistic regression analysis:** the socio-demographic and clinical factors noted to be significantly associated with malaria parasitaemia were further analyzed using binary logistic regression analysis. Low social class (OR 2.457; 95% CI 1.404 - 4.300; p=0.002), antenatal care at maternity centres/PHCs (OR 2.226; 95% CI 1.096 - 4.522; p=0.027), low birth weight (OR 4.818; 95% CI 2.317 - 10.018; p=<0.001) and use of haematinics (OR 3.192; 95% CI 1.731 - 5.886; p=<0.001) were independent predictors of the presence of malaria parasitaemia among the infants (Table 4).

**Table 4:** predictors of malaria parasitaemia using multivariate logistic regression

Parameters	B	SE	Odd ratio	95% CI of Odd ratio	p-value
Low social class	0.899	0.286	2.457	1.404-4.300	0.002
Residence outside Ilesha	-0.642	0.342	0.526	0.269-1.028	0.060
Non-use of IPT	-0.604	0.317	0.546	0.293-1.018	0.057
ANC at maternity centres/PHC	0.800	0.362	2.226	1.096-4.522	0.027
ANC in other hospitals	0.105	0.416	1.110	0.492-2.507	0.801
Delivery at maternity centres/PHC	0.279	0.402	1.322	0.601-2.905	0.488
Lack of ANC	-1.105	0.572	0.331	0.108-1.017	0.054
Low birth weight	1.572	0.373	4.818	2.317-10.018	<0.001
Exclusive breastfeeding	-0.468	0.372	0.627	0.302-1.299	0.209
Non-use of window nets	0.778	1.277	0.459	0.038-5.614	0.459
Use of haematinics	1.161	0.312	3.192	1.731-5.886	<0.001

B: coefficient of regression; SE: standard error; Cl: confidence interval

## Discussion

This study found significant prevalence of malaria parasitaemia among young infants who were believed to be protected from malaria infection with some of them having heavy malaria parasite densities. It also highlights the identified risk factors for malaria infection among the study participants. The prevalence rate of malaria parasitaemia among the infants, aged one to six months recruited for this study was 19.1%. This was similar to 21.7% reported by Ceesay *et al*. [9] among young infants in Guinea, a West African country with high malaria transmission intensity like Nigeria. Ceesay *et al*. [9] included neonates and could have recruited some of the neonates with congenital malaria. Malaria detection rate in the present study was greater than 10.3% reported from Benin city, Nigeria [4], 10.5% from Democratic Republic of Congo [20], 3.7% from the Gambia [9] and 10.2% from Benin Republic [9]. The observed higher prevalence of malaria parasitaemia in this study compared to aforementioned studies may be related to the recruitment of ill infants as compared to apparently healthy infants in the aforementioned studies. Brazeau *et al*. [20] in the Democratic Republic of Congo used a more specific malaria diagnostic tool (polymerase chain reaction) to detect the malaria parasite unlike this study that used microscopy. The lower malaria parasitaemia rate in young infants reported from The Gambia and Benin Republic may be due to the low and moderate malaria transmission intensity in these countries compared to what obtains in Nigeria with a high malaria transmission intensity [9, 21].

In contrast to this present study, Afolabi *et al*. [8] in Lagos, Nigeria reported a much higher prevalence of 27.1% for malaria parasitaemia among the young infants recruited for their study. The infants were in their first six months of life including neonates. The study was conducted in a densely populated part of Lagos state with poor social amenities and environmental sanitation while Ilesa is less densely populated and crowded. Also, unlike the present study, neonates were included in the study by Afolabi *et al*. [8] possibly some cases of congenital malaria could have been included in their study.

Most studies have reported predominantly low parasite density among infants with malaria parasitaemia [8, 9]. However, about one in four of the infants with malaria infection in this study had heavy malaria parasitaemia. This significant finding may be due to the inclusion of very ill infants from the children emergency ward where infants with heavy parasitaemia will likely present. It may also be a pointer to the changing trend in the magnitude of the challenge of malaria infection among this group of infants [8, 21]. Parental low socio-economic class was a predictor of the presence of malaria parasitaemia in ill infants recruited for this study. Majority of the countries with high prevalence of malaria are the ones with high level of poverty [22, 23]. This finding was also corroborated by Tusting *et al*.[24] in rural Uganda who also observed significant association between parental low socioeconomic status and increased incidence of malaria parasitaemia among infants. Parents from the low socioeconomic class may have poor housing conditions [24] (for example absence of windows), puddles of water and poor water drainage around the house and these are breeding sites for mosquitoes. Hence, efforts to reduce the burden of malaria in Africa and similar endemic region should be holistic to include poverty alleviation and not just the provision of antimalarial drugs or malaria vaccine.

Mothers from the low socioeconomic class may not be able to afford quality antenatal care during pregnancy [25]. Therefore, low social class and poor antenatal care are closely related. Corroborating this, is the finding in this study that a higher proportion of infants who had malaria parasitaemia belonged to mothers who delivered outside the hospital facility (home and spiritual birth homes) compared to those without malaria parasitaemia. Contrary to the finding in this study, Onwujekere *et al*. [26] in Anambra, Nigeria reported that there were fewer reports of malaria among the rural dwellers and those in the low social class compared to individuals who were residing in the urban centres and those of the high social class. The reason for this difference in the findings from the present study and that of Onwujekere *et al*. [26] because this present study used the level of education, type of occupation and the income of parents to categorize the infants into different socioeconomic groups while Onwujekere *et al*. [26] used less specific social class criterion i.e. the number of properties the household possessed as the basis for socioeconomic class placement. Infants recruited for this present study had laboratory confirmation of malaria while the latter used the less accurate self-report of fever and treatment for malaria in the preceding one month before the study as their diagnostic criteria.

Antenatal care (ANC) in maternity centres or PHC (which are sometimes privately owned), was associated with higher proportion of infants with malaria parasitaemia compared with those without malaria parasitaemia. Poor quality of care was one of the reasons why pregnant women in a rural area of Edo State would rather not access antenatal care at their primary health centre as reported by Okonofua *et al*. [27]. The WHO includes competent health care providers as an important component of effective antenatal care package [28]. Most PHCs in Nigeria are manned by community health/extension workers with little or no input from more qualified health workers [27]. This can adversely affect service delivery including ANC at these PHCs. On the other hand, most tertiary health centres (like the WGH) provide these basic ANC services and often times even more for the pregnant women that access such care in the facilities. IPT use during pregnancy is an important component of ANC because it offers protection for both the mothers and their young infants. This is because IPT reduces placental parasitisation with malaria parasite which has been linked to higher risk of malaria parasitaemia early in infancy [10]. However, Fagbamigbe *et al*. [29] reported that IPT was among the least antenatal care services received by pregnant mothers in a national survey on the quality of antenatal care received by pregnant women in Nigeria. The study also reported that the highest proportion of those who did not have IPT and other basic ANC was among those that registered at maternity centres especially the private maternity homes. Likewise, non-use of IPT during pregnancy was observed to be significantly associated with increased risk of malaria parasitaemia among the infants in this study. Although, the positive effect of IPT in pregnancy and by extension the infant has been challenged with the low coverage of IPT use in Nigeria and emerging resistance to the IPT drug [10].

Infants on routine haematinics (iron and folate supplements) were three times more likely to have malaria parasitaemia compared to those who were not on any supplement. This is similar to the findings of Gwamaka *et al*. [30] that iron deficiency reduced the prevalence of malaria parasitaemia and even the mortality from malaria infection among a cohort of Tanzanian children. However, Zlotkin *et al*. [31] in a randomized trial conducted in Ghana reported an overall lower incidence of malaria cases and anaemia among children who were on iron supplement compared to those who were on non-iron containing supplements. Although additional interventions like the use of insecticide treated nets and appropriate malaria treatment were ensured in both groups in the Zlotkin *et al*. [31] study this probably accounted for the outcome reported. Relative iron deficiency may offer some form of protection against severe malaria infection (and even other pathogenic organisms) in children residing in sub-Saharan Africa where malaria is prevalent [30]. Iron deficiency however has negative effects like severe anaemia and poor cognitive development in the infants [21]. Therefore, WHO [32] recommends that iron supplementation in malaria endemic regions for children can be done when adequate malaria prevention (use of mosquito nets, use of insecticide etc.) and treatment have been made available. This is to prevent an increase in cases of malaria especially the severe forms.

Low birth weight babies were about five times more likely to have malaria parasite infection compared to other infants who had normal weight at birth. This is because parasitization of the placental by malaria parasite has been strongly associated with low birth weight [7]. Studies have also reported that infants born as low birth weight due to malaria infection in their mothers are at increased risk of having malaria infection early in infancy [23]. This increased susceptibility to malaria infection has been associated with reduced antibody transfer from the pregnant mother with malaria infection to her foetus [5, 7] and also tolerance of the infant´s immunity to the malaria parasite that the infant might have been exposed to in utero [7].

Non-use of window nets was significantly associated with presence of malaria parasitaemia. While other WHO malaria control strategies like the use of insecticide treated bed-nets, indoor spraying of insecticides and use of mosquito nets on the doors were not significantly related to malaria parasitaemia. This finding may result from the high utilization rate (89.7%) of window nets recorded in this study compared to the use of insecticidal treated bed-nets (48.6%), use of indoor insecticide spray (40.3%) and presence of mosquito net on the main door (29.1%). This study was hospital based and may not be a true reflection of what obtains in the community.

The adequate sample size, carefully selected homogenous study population and laboratory confirmation of malaria are strengths of our study. We however appreciate the fact that we couldn´t further confirm malaria parasitaemia using more accurate polymerase chain reaction methods which is devoid of the individual differences in the use of microscopy. Nevertheless, this study has highlighted the burden, sociodemographic and perinatal predictors of the presence of malaria parasitaemia among ill young infants in an endemic region who otherwise should be protected from this infection.

## Conclusion

Malaria infection was believed to be uncommon among young infants. This study was therefore designed to determine the prevalence, parasite density and the risk factors associated with malaria parasitaemia in the young infants presenting with ill-health at the children welfare clinic (CWC) and children emergency ward (CEW) of the Wesley Guild Hospital (WGH), Ilesa, south-west Nigeria.

The prevalence of malaria parasitaemia among the ill young infants in this study was 19.1% (i.e about one of five of the ill infants had malaria parasitaemia). Also, about a quarter of the infants infected had heavy malaria parasite densities. Identified predictors of malaria parasitaemia include infants who were low birth weight and had haematinics (iron and folic acid supplements). Others were parental low socioeconomic class and those whose mothers had sub-standard antenatal care.

Therefore, health workers should have high index of suspicion for malaria infection in this age group particularly the LBW infants, those on iron and folic acids supplements, and those from low socioeconomic class, whose mothers received substandard ANC. Also, ensuring universal access to quality and affordable maternal and child care services will assist in reducing the burden of malaria parasitaemia among these infants. Economic policies should be put in place by the government to improve the socioeconomic status of the populace.

### What is known about this topic

- Malaria infection among young infants is uncommon;
- There are identified protective mechanisms against malaria infection in young infants;
- Light malaria parasitaemia is common among those infected.

### What this study adds

- Malaria infection does occur in young infants; one in five of the ill young infants at the Wesley Guild Hospital, Ilesa had malaria parasitaemia;
- Young infants with malaria parasitaemia can have heavy parasite density, about twenty-five percent of those infected had heavy malaria parasitaemia in this study;
- Low social class, sub-optimal antenatal care for the pregnant mothers, being low birth weight and indiscriminate use of haematinics were independent predictors of malaria parasitaemia among the infants.
